# Evaluating Laparoscopic Simulation Training for Preclinical Osteopathic Medical Students

**DOI:** 10.7759/cureus.103231

**Published:** 2026-02-08

**Authors:** Ryan D Muchard, Nathan D Jacobsen, Tristan Sypula, Sawyer Longley, Nikolete Hurrinus, Natalie S Barefield, Praful G Patel

**Affiliations:** 1 Research Department, Alabama College of Osteopathic Medicine, Dothan, USA

**Keywords:** competency, laparoscopic simulation training, laparoscopy, medicaleducation, medical education, minimally invasive surgery, simulation, simulation in medical education, surgical education, surgical training

## Abstract

Objective

To assess the effectiveness of a laparoscopic simulation training program using LAPARO medical simulators in enhancing the surgical skills competency and confidence of osteopathic medical students.

Background

Pre-clinical education significantly influences the professional development of future physicians. Early exposure to laparoscopic techniques can help medical students explore surgical interests and develop skills for clerkships and future careers.

Methods

Participants performed three LAPARO simulation modules: Beads, Cones, and Pegs. Their best times from two attempts each in February and May 2024 were recorded. Attempts exceeding four minutes were classified as failures. Participants attended multiple practice sessions between assessments. In addition, a Qualtrics survey comprising nine questions was administered to evaluate interest in surgery, perceived skill development, and attitudes toward laparoscopic training.

Results

The study found that 83.33% of participants improved in at least one module. There was a 62.1% reduction in failure rates from initial to final assessments. In the Beads module, the initial failure rate was 100%, decreasing to 66.67% with a final average time of 3:11, a 34% reduction. For the Cones module, the initial failure rate was 47.37%, improving to 16.67% with an average time decrease from 2:59 to 2:31 (15.65% improvement). In this module, 75% improved their times, and three participants achieved times under two minutes. The Pegs module had an initial failure rate of 5.26%, which increased slightly to 8.33%, but the average time improved from 2:34 to 2:27 (4.55% reduction). Sub-two-minute completions increased from 15.79% to 33.33%, with 58.33% improving their times. Significant improvement was observed in the Cones module (p = 0.0036), with overall trends across all modules suggesting enhanced performance following practice, reinforcing the educational impact of laparoscopic simulation training. Survey data revealed strong positive perceptions: 93.2% of respondents agreed that the laparoscopic events increased their interest in laparoscopic surgery, 81.8% reported greater interest in general surgery, and 81.9% felt more comfortable with their laparoscopic skills for clinical rotations.

Conclusion

Objective performance improvements and positive survey responses collectively demonstrate that early laparoscopic simulation training may enhance technical proficiency, confidence, and enthusiasm for surgical careers among pre-clinical osteopathic medical students. These findings support the early integration of simulation into pre-clinical medical education and highlight its dual role in developing skills and fostering surgical interest. Further research is needed to explore the long-term benefits and potential applications of such training.

## Introduction

Minimally invasive surgery (MIS) has become a foundational technique across numerous surgical specialties, offering patients reduced postoperative pain, shorter hospital stays, and faster recovery. As such, laparoscopic and robotic procedures have become standard in many clinical settings, with a 2020 study reporting that approximately 93% of appendectomies were performed laparoscopically [1]. Given the increasing prevalence of MIS, both laparoscopy and robotics, surgical trainees are expected to develop competency in laparoscopic skills early in their education.

To meet this demand, the fundamentals of laparoscopic surgery (FLS) examination has become a requirement for board certification in both general surgery and obstetrics and gynecology [2,3]. Simulation-based training has emerged as an effective method for acquiring these technical skills. In a multi-institutional survey of surgical residents at Yale University and the University of Toronto, 95.5% of respondents reported improvements in their laparoscopic skills with simulation training, and 92.5% reported that these skills were transferable to the operating room [4].

Early exposure to laparoscopic simulation provides medical students with a valuable opportunity to develop foundational skills prior to entering clinical clerkships. This hands-on experience not only supports skill acquisition but also allows students to explore surgical interests and improve their preparedness for the operating room environment. In settings where access to high-volume surgical centers may be limited, simulation may serve as a critical tool in bridging the training gap.

This study aims to assess the effectiveness of a student-run laparoscopic simulation training program using LAPARO® medical simulators in enhancing the surgical competency and interest of preclinical osteopathic medical students. By evaluating objective performance metrics across multiple simulation modules alongside subjective survey data on surgical interest and confidence, this study explores the value of early simulation exposure in surgical education and its potential impact on future training outcomes.

## Materials and methods

**Figure 1:**
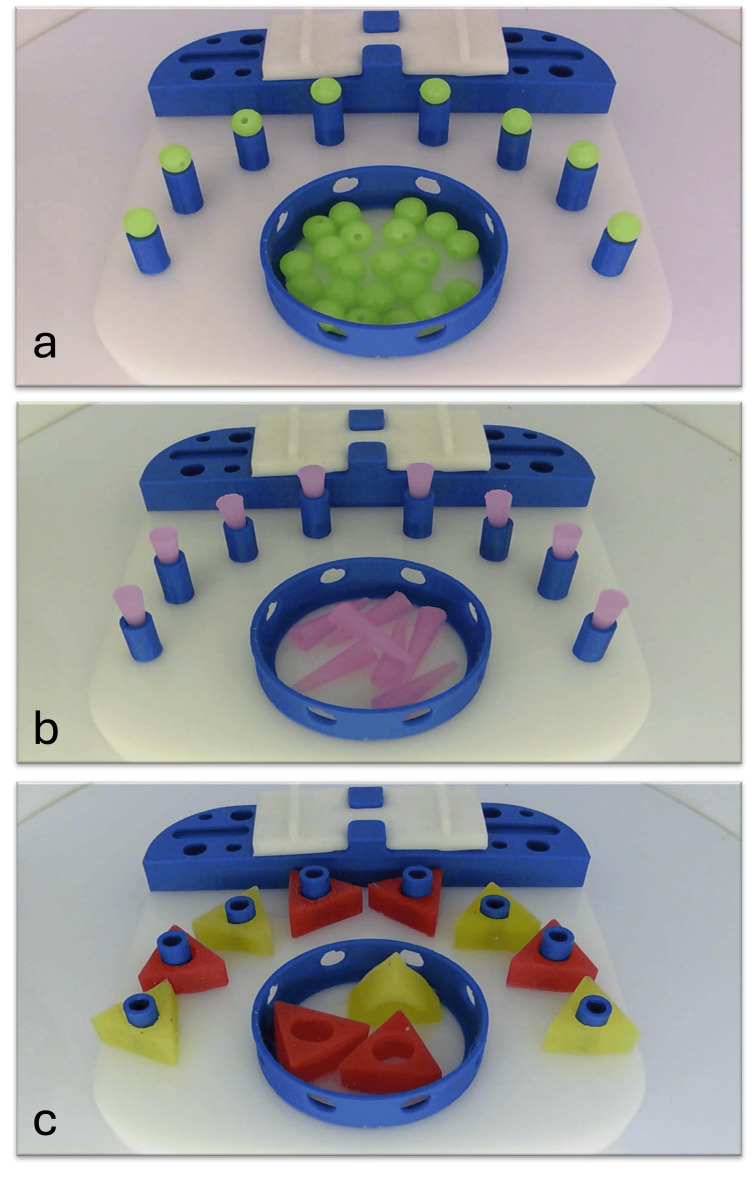
Laparoscopic Modules a - Beads module; b - Cones module; c - Pegs module

This study assessed the impact of laparoscopic simulation training on pre-clinical osteopathic medical students (OMS-I and OMS-II) at the Alabama College of Osteopathic Medicine. The training utilized LAPARO® medical simulators and consisted of three standardized modules: Beads, Cones, and Pegs (Figure 1). The LAPARO® system is a portable, box-based laparoscopic simulator that uses standard laparoscopic instruments and camera visualization. Each module was designed to assess and develop core laparoscopic skills, including depth perception, hand-eye coordination, and precision with instrument handling. Population of first- and second-year osteopathic medical students (OMS-I and OMS-II) at the Alabama College of Osteopathic Medicine with limited or no prior laparoscopic experience (n=19).

Simulation tasks were loosely based on the FLS framework but adapted for preclinical learners, with emphasis on foundational skills rather than formal FLS training or certification. Using a fenestrated grasper and a Maryland dissector in their opposite hand, students were instructed to grasp each object from the central basin and transfer it mid-air to the opposite hand. The object was then placed on any available peg on the board. No emphasis was placed on the color or order of the objects during the transfer process. All transfers were required to be performed mid-air, without using the board or pegs for support.

Participants were permitted two attempts per module during each testing session. The fastest of the two attempts for each participant was used to calculate the final average for each module. Attempts exceeding four minutes were classified as failures. Baseline assessments were conducted in February 2024, with follow-up assessments completed in May 2024, approximately four months apart.

Between these two assessments, students were provided the opportunity to attend up to seven open lab practice sessions. These sessions enabled participants to engage in self-directed practice using the LAPARO simulators under informal faculty supervision. Attendance was encouraged but not mandatory, and participants were not provided with individualized instruction during assessment attempts.

In addition to performance metrics, the authors developed a web-based survey utilizing the Qualtrics XM platform (Qualtrics, North Sydney, Australia) that was administered to participants to assess interest, confidence, and perceptions of laparoscopic training. The survey included nine questions covering demographics, event attendance, interest in surgical careers, and self-perceived skill development. Responses were collected using multiple-choice and Likert-scale formats. A total of 44 survey responses were collected from the 19 participants across the seven sessions, reflecting repeated responses from individual participants over time.

Statistical analysis

Performance improvement was evaluated by comparing initial and final average completion times and failure rates across the three modules. The primary outcome measure was the proportion of students who demonstrated improvement in at least one module. Secondary outcomes included module-specific changes in failure rates, average completion times, and the proportion of participants achieving sub-two-minute completions. Paired t-tests were conducted to compare initial and final attempt performance metrics to assess for statistically significant improvements across modules. Participants with failed attempts were excluded from paired t-tests to ensure comparison of continuous completion-time data only, as failed attempts were censored at the predefined four-minute threshold and therefore not directly comparable to completed trials. Survey responses were analyzed using descriptive statistics to examine survey categories, including surgical career interest, perceived skill comfort, and overall perception of surgery.

## Results

Nineteen osteopathic medical students participated in the initial assessment (n=19), with 12 completing the final assessment (n=12), resulting in a 63.16% retention rate. Each participant completed two attempts on three LAPARO simulation modules - Beads, Cones, and Pegs - with the best time recorded. Times exceeding four minutes were considered failures. Overall, 10 (83.33%) of the returning participants demonstrated improvement in at least one module. All task completion times are reported in minutes:seconds (mm:ss), unless otherwise specified.

For the Beads module at baseline, 19 (100%) students failed to complete the Beads module within the four-minute limit. At the final assessment, the failure rate decreased to 8 (66.67%), representing a 34% reduction. The average time for the final session was 3:11. Four students (33.33%) improved their completion times across sessions.

The initial failure rate for the Cones module was 9 out of 19 (47.37%), with an average completion time of 2:59. At the final assessment, the average time improved to 2:31, reflecting a 15.65% reduction in average time. The failure rate dropped to two students out of 12 (16.67%), indicating a 30.70% improvement. Nine (75%) students improved their times, and three (25%) students completed the module in under two minutes, compared to none at baseline.

**Figure 2:**
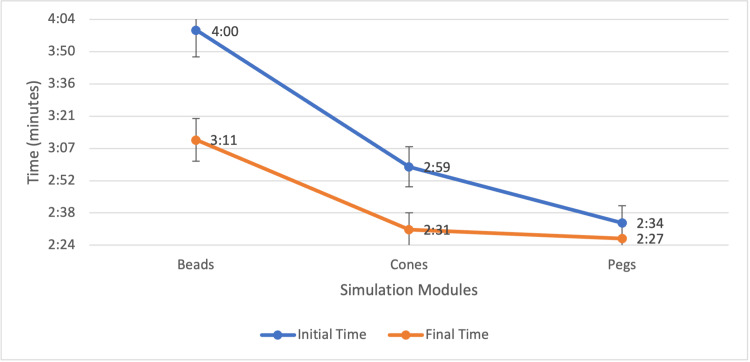
Improvement in the Average Completion Times Across the Simulation Modules Line graph depicting average initial and final completion times (in minutes:seconds) for each laparoscopic simulation module: Beads, Cones, and Pegs. Following the training intervention, average times improved across all three modules. The initial average time for the Beads module exceeded the four-minute failure threshold (denoted at 4:00), while final times for all modules were below this benchmark. Data labels indicate average times at each timepoint. Final times demonstrate consistent improvement, particularly in the Beads and Cones modules.

**Figure 3:**
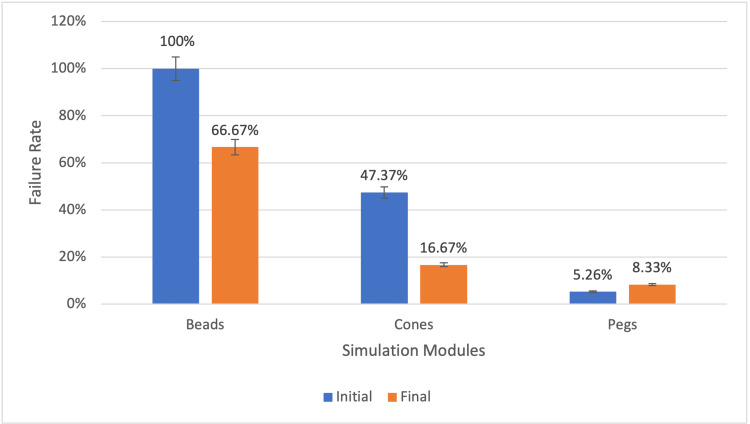
Initial vs Final Failure Rates by Module Bar graph showing the percentage of participants failing to complete each module within four minutes at the initial and final assessments. Percentages are used to facilitate relative comparison between timepoints despite differences in participant numbers.

The Pegs module exhibited a low failure rate at both baseline and final testing, with one out of 19 (5.26%) and one out of 12 (8.33%) failures, respectively. However, average completion time improved from 2:34 to 2:27. The proportion of sub-two-minute completions increased from three students (15.79%) to four students (33.33%), a 17.54% improvement. Seven participants (58.33%) improved their performance (Figures 2, 3).

Participants were stratified to include only those who successfully completed both the initial and final timing sessions without task failure (Cones: n=10; Pegs: n=11) for paired comparisons of task completion times (Table 1). Two-tailed paired t-tests demonstrated a statistically significant reduction in completion time for the Cones module (p=0.0036), indicating improved performance following repeated practice. No significant difference was observed for the Pegs module (p=0.756). Statistical analysis for the Beads module was not conducted due to a high number of failed attempts and insufficient paired data (Table 2). Despite limited sample sizes and not all modules meeting the threshold for statistical significance, the overall trend demonstrated consistent improvement across modules, supporting the educational value of laparoscopic simulation training.

**Table 1:** Raw Task Completion Times for Participants With Successful Initial and Final Attempts Raw task completion times (seconds) for participants who successfully completed both the initial and final timing sessions without task failure. Data are shown for the Cones (n=10) and Pegs (n=11) laparoscopic simulation modules. Only participants with complete paired data were included in subsequent paired statistical analyses. All times were recorded in seconds to facilitate direct comparison and statistical testing.

Participant Number	Initial time cones (sec)	Final time cones (sec)	Initial time pegs (sec)	Final time pegs (sec)
1	240	230	150	233
2	213	170	196	197
3	152	127	72	70
4	204	192	98	112
5	150	110	127	113
6	240	145	180	133
7	134	118	66	95
8	240	165	179	124
9	132	110	239	120
10	240	224	135	196
11	-	-	240	230

**Table 2:** Paired T-test Results Comparing the Initial and Final Task Completion Times Across Laparoscopic Modules Two-tailed paired t-tests were performed to compare initial and final task completion times for participants who successfully completed both sessions for each laparoscopic training module. A statistically significant improvement was observed in the Cones module (p=0.0036). No significant difference was found for the Pegs module (p=0.756). Statistical analysis for the Beads module was not performed due to a high number of failed attempts and insufficient paired data points (n=4 successful final attempts). The corresponding t-values and degrees of freedom (df) are included in the table to provide additional context for the statistical analysis.

Module	Initial mean time (sec)	Initial standard deviation (sec)	Final mean time(sec)	Final standard deviation (sec)	p-value	T-value	df	Sample size (n)
Cones	194.5	47.00	159.1	44.00	0.0036	3.89	9	10
Pegs	152.9	60.00	147.5	56.00	0.756	0.32	10	11
Beads	-	-	191	18	N/A	N/A	N/A	4

Survey results demonstrated a positive influence of laparoscopic simulation events on student interest and confidence. Among respondents, 70.5% indicated an interest in a surgical career, while 22.7% were undecided. Overall, 93.2% agreed that the events increased their interest in laparoscopic surgery, and 81.8% reported increased interest in general surgery. Additionally, 81.9% reported greater comfort with their laparoscopic skills for upcoming surgical rotations, and 95.5% reported that participation positively influenced their perception of the field of surgery.

## Discussion

This study demonstrates that a student-run laparoscopic simulation training program can enhance the technical proficiency of preclinical osteopathic medical students. The majority of participants demonstrated measurable improvement in at least one module, with notable reductions in failure rates and average task completion times, particularly in the Bead and Cone modules. These results support the integration of early simulation-based training into undergraduate medical education as a means to strengthen foundational surgical skills.

Although the differences in completion time between the initial and final simulation attempts did not all reach statistical significance, the observed improvements across all modules suggest a meaningful educational trend. These results suggest that participation in the simulation program may still offer value in developing laparoscopic skills; the lack of statistical significance in performance metrics is likely due to the small sample size.

These findings reinforce the growing consensus that structured simulation is a critical adjunct to traditional surgical training [5-8]. Multiple systematic reviews and meta-analyses have shown that simulation, whether delivered through box trainers, virtual reality, or low-cost alternatives, substantially improves technical performance, knowledge retention, and error reduction compared to conventional training alone [9-13]. Introducing simulation during the preclinical years has been shown to accelerate skill acquisition, increase confidence, foster student enthusiasm and preparedness for clinical clerkships, and spark interest in surgical careers [14-16].

The success of this student-led program also highlights the feasibility of implementing low-resource simulation curricula at institutions without large hospital affiliations. Prior studies suggest that early exposure to surgical skills and operating room principles can positively influence perceptions of surgery and support workforce development efforts [5,10,11,14,15].

The inclusion of survey data reinforces the educational value of laparoscopic simulation beyond technical skill development. The overwhelmingly positive responses, notably the 93% of students who reported increased interest in laparoscopy and the 96% who noted a more favorable perception of surgery, underscore the motivational and career-influencing potential of early exposure. These findings align with prior literature suggesting that hands-on simulation enhances both confidence and enthusiasm for surgical fields, which may contribute to improved match rates into surgical specialties [16].

Although clinical performance was not directly assessed, improvements in task efficiency and reductions in failure rates observed in this study may reflect early development of foundational laparoscopic skills such as hand-eye coordination, depth perception, and instrument handling. Prior educational literature suggests that early acquisition of these skills may help mitigate the initial learning curve encountered during surgical clerkships, potentially enhancing learner readiness and confidence during clinical training [11-13].

This study is not without limitations. Conducted at a single institution with a small sample size and a 63.16% retention rate, the results may not be broadly generalizable. Skill retention over time and translation to clinical performance were not assessed. Additionally, attendance at optional practice sessions was not standardized, potentially introducing variability in student performance. Furthermore, the single-institution design may reflect institutional culture or curricular factors that are not representative of other training environments. These challenges mirror broader issues in simulation research, including variable curriculum integration, lack of long-term follow-up, and logistical barriers to scalability [17-19].

Future directions should include multicenter studies with larger and more diverse student populations, as well as longitudinal follow-up to assess the durability of skill acquisition and its impact on clinical outcomes. Development of standardized outcome metrics and cost-effectiveness analyses will be important for informing best practices and optimizing resource allocation. To expand accessibility, future efforts should also explore the use of low-cost and mobile simulation platforms, particularly in underserved training environments [18-20].

## Conclusions

This study demonstrates that a student-led laparoscopic simulation program can enhance technical proficiency and promote early skill development among preclinical osteopathic medical students. Statistical analyses reveal significant differences in cone module completion times and consistent trends toward improvement across all modules, highlighting the educational value of structured simulation, particularly for institutions without large surgical centers. Importantly, the observed reduction in failures and improvements in task efficiency suggest that, even in a small sample size, early exposure to minimally invasive surgical techniques can foster student confidence, prepare learners for clerkships, and encourage interest in surgical careers. In addition to measurable performance improvements, survey findings from simulation sessions indicate that early laparoscopic simulation experiences significantly enhance students' interest, confidence, and perceptions of surgery as a career. These findings underscore the potential role of simulation as a practical and accessible adjunct to traditional medical education, supporting the further implementation and expansion of such a curriculum in preclinical training environments.

## Figures and Tables

**Figure 4:**
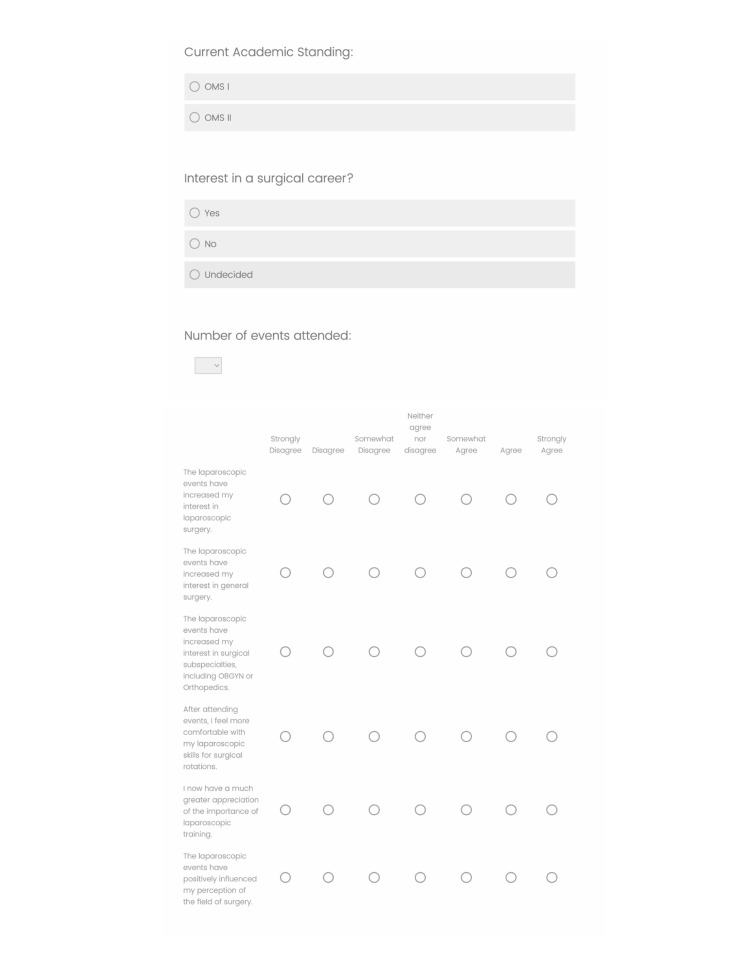
Qualtrics Survey Qualtrics survey instrument used to assess medical student demographics, prior exposure to laparoscopic simulation, perceived knowledge, interest in surgical careers, and attitudes toward early laparoscopic skill development. The survey consisted of nine questions utilizing multiple-choice, drop-down, and Likert-scale formats.

## Appendices

Figure 4 is the Qualtrics survey made.

## References

[1] St John A, Caturegli I, Kubicki NS, Kavic SM (2020). The rise of minimally invasive surgery: 16 year analysis of the progressive replacement of open surgery with laparoscopy. JSLS.

[2] Hur HC, Arden D, Dodge LE, Zheng B, Ricciotti HA (2011). Fundamentals of laparoscopic surgery: a surgical skills assessment tool in gynecology. JSLS.

[3] Yochim MN, Yang H, Apostol R (2020). Fundamentals of laparoscopic surgery exam: a cross-sectional survey of in-training obstetricians and gynecologists. J Minim Invasive Gynecol.

[4] Shetty S, Zevin B, Grantcharov TP, Roberts KE, Duffy AJ (2014). Perceptions, training experiences, and preferences of surgical residents toward laparoscopic simulation training: a resident survey. J Surg Educ.

[5] Zhang W, Qu L, Zilundu PL (2025). Advancing surgical education: an evaluation of laparoscopic simulation training for medical students. J Eval Clin Pract.

[6] Taba JV, Cortez VS, Moraes WA (2021). The development of laparoscopic skills using virtual reality simulations: a systematic review. PLoS One.

[7] Zendejas B, Brydges R, Hamstra SJ, Cook DA (2013). State of the evidence on simulation-based training for laparoscopic surgery: a systematic review. Ann Surg.

[8] Awal W, Dissabandara L, Khan Z, Jeyakumar A, Habib M, Byfield B (2021). Effect of smartphone laparoscopy simulator on laparoscopic performance in medical students. J Surg Res.

[9] Sellers T, Ghannam M, Asantey K, Klei J, Olive E, Roach VA (2021). An early introduction to surgical skills: validating a low-cost laparoscopic skill training program purpose built for undergraduate medical education. Am J Surg.

[10] De Win G, Van Bruwaene S, Aggarwal R, Crea N, Zhang Z, De Ridder D, Miserez M (2013). Laparoscopy training in surgical education: the utility of incorporating a structured preclinical laparoscopy course into the traditional apprenticeship method. J Surg Educ.

[11] Al-Kadi AS, Donnon T (2013). Using simulation to improve the cognitive and psychomotor skills of novice students in advanced laparoscopic surgery: a meta-analysis. Med Teach.

[12] Al-Kadi AS, Donnon T, Oddone Paolucci E, Mitchell P, Debru E, Church N (2012). The effect of simulation in improving students' performance in laparoscopic surgery: a meta-analysis. Surg Endosc.

[13] Sarmiento-Altamirano D, Ormaza F, Arroyo MR, Cabrera-Ordoñez C, Valdivieso R, Docksey M, Di Saverio S (2024). Optimizing laparoscopic and robotic skills through simulation in participants with limited or no prior experience: a systematic review and meta-analysis. J Gastrointest Surg.

[14] Palenzuela D, Pradarelli A, McKinley S, Moses J, Saillant N, Phitayakorn R (2023). Preclinical immersion experiences improve medical student perceptions of surgery. J Surg Res.

[15] Gawad N, Zevin B, Bonrath EM, Dedy NJ, Louridas M, Grantcharov TP (2014). Introduction of a comprehensive training curriculum in laparoscopic surgery for medical students: a randomized trial. Surgery.

[16] Lazow SP, Venn RA, Lubor B (2019). The PreOp program: intensive preclinical surgical exposure is associated with increased medical student surgical interest and competency. J Surg Educ.

[17] Acton RD (2015). The evolving role of simulation in teaching surgery in undergraduate medical education. Surg Clin North Am.

[18] Sparn MB, Teixeira H, Chatziisaak D, Schmied B, Hahnloser D, Bischofberger S (2024). Virtual reality simulation training in laparoscopic surgery - does it really matter, what simulator to use? Results of a cross-sectional study. BMC Med Educ.

[19] Bjerrum F, Thomsen AS, Nayahangan LJ, Konge L (2018). Surgical simulation: current practices and future perspectives for technical skills training. Med Teach.

[20] Abahuje E, Tuyishime E, Alayande BT (2025). Global surgical simulation education, current practices, and future directions. Surgery.

